# Supplementary material for: SENP3 maintains the stability and function of regulatory T cells via BACH2 deSUMOylation
Source: Nat Commun. 2018 Aug 8;9:3157. doi: 10.1038/s41467-018-05676-6 (PMC6082899; doi:10.1038/s41467-018-05676-6)
Supplement: Supplementary file 1 — Supplementary Information [file 41467_2018_5676_MOESM1_ESM.pdf]

SENP3 maintains the stability and function of regulatory T cells via BACH2 deSUMOylation

Yu et al.

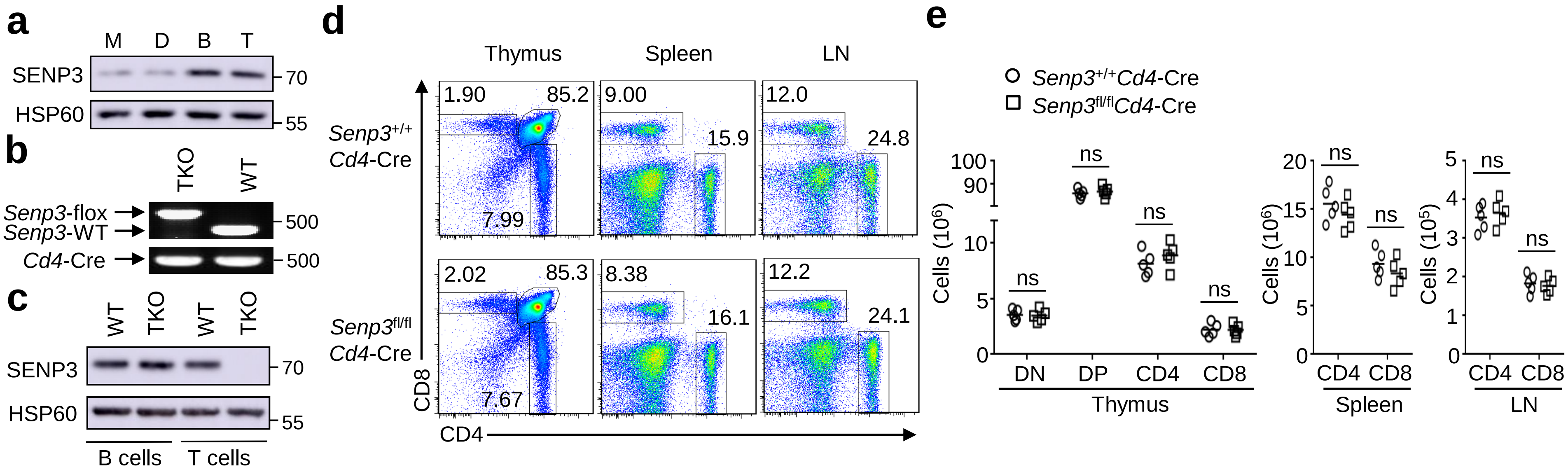

Supplementary Figure 1. Thymocyte development and peripheral T cell frequency in the *Senp3*$^{fl/fl}$*Cd4*-Cre mice. (**a**) Immunoblot analysis of SENP3 in whole-cell lysates of bone marrow-derived macrophages (M), bone marrow-derived DCs (D), splenic B cells (B) and splenic T cells (T). (**b**) Genotyping PCR to amplify the *Senp3*-flox and WT alleles (top) or the Cre cDNA (bottom). (**c**) Immunoblot analysis of SENP3, showing T cell–specific SENP3 ablation (WT: *Senp3*$^{+/+}$*Cd4*-Cre; TKO: *Senp3*$^{fl/fl}$*Cd4*-Cre) . (**d**) Flow cytometric analysis of the percentage of $CD4^+$ and $CD8^+$ T cells from the thymus, spleen and lymph nodes (LN) of 6-week-old *Senp3*$^{+/+}$*Cd4*-Cre and *Senp3*$^{fl/fl}$*Cd4*-Cre mice. (**e**) Cell number of $CD4^-CD8^-$ (DN), $CD4^+CD8^+$ (DP), $CD4^+CD8^-$ (CD4), $CD4^-CD8^+$ (CD8) in the thymus, and $CD4^+$ and $CD8^+$ T cells in the spleen and LN of 6-week-old *Senp3*$^{+/+}$*Cd4*-Cre and *Senp3*$^{fl/fl}$*Cd4*-Cre mice. Data are representative of at least three independent experiments. Error bars are the mean $\pm$ SEM values. n = 5. Two-tailed unpaired Student's *t* tests were performed. ns, not statistically significant.

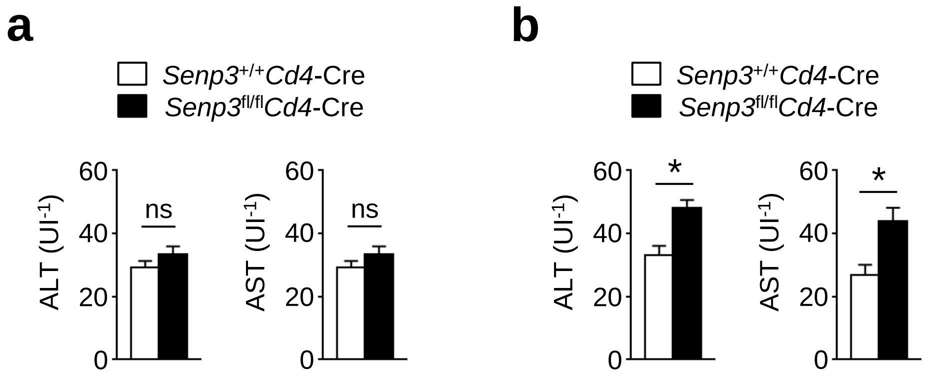

Supplementary Figure 2. Serum AST and ALT concentrations of the *Senp3*$^{fl/fl}$*Cd4*-Cre mice. (**a**) Serum AST and ALT concentrations of 8-week-old *Senp3*$^{+/+}$*Cd4*-Cre and *Senp3*$^{fl/fl}$*Cd4*-Cre mice. (**b**) Serum AST and ALT concentrations of 8-month-old *Senp3*$^{+/+}$*Cd4*-Cre and *Senp3*$^{fl/fl}$*Cd4*-Cre mice. Data are representative of at least three independent experiments. Error bars are the mean ± SEM values. n = 5. Two-tailed unpaired Student's *t* tests were performed. ns, not statistically significant. *, $P<0.05$.

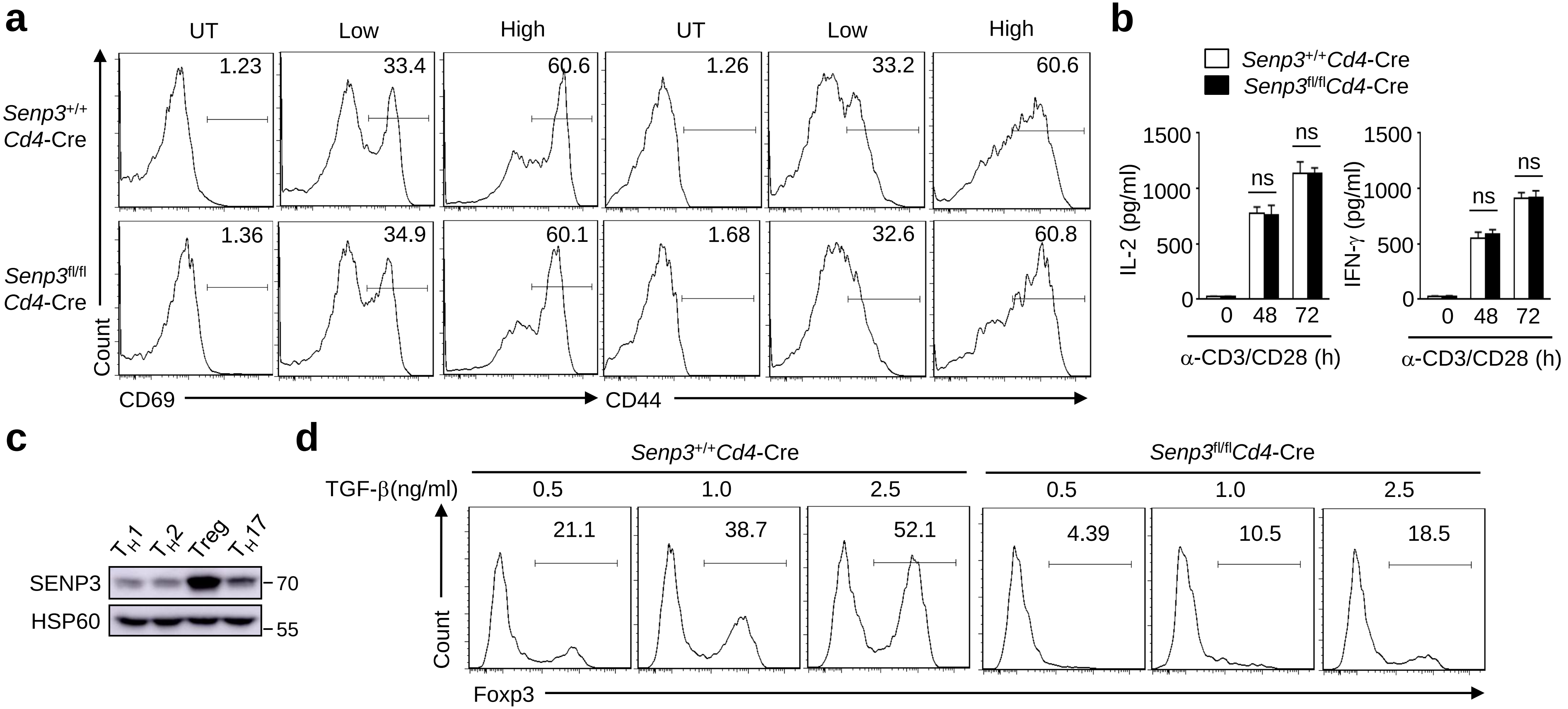

Supplementary Figure 3. T cell activation and Treg cell differentiation of naive *Senp3*$^{fl/fl}$*Cd4*-Cre CD4$^+$ T cells. (**a**) Flow cytometric analysis of CD69 and CD44 in naive CD4$^+$ T cells stimulated for 24 h with the different doses of anti-CD3 and anti-CD28.UT, unstimulated; Low, 0.25μg/ml anti-CD3 and anti-CD28; High,1.0μg/ml anti-CD3 and anti-CD28.(**b**) ELISA of secreted IL-2 and IFN-γ of naive CD4$^+$ T cells either not treated (0) or stimulated for indicated amounts of time. (**c**) Immunoblot analysis of SENP3 in various T cell subsets that were differentiated *in vitro*. (**d**) Flow cytometric analysis of Foxp3 expression in naive CD4$^+$ T cells (CD4$^+$CD44^{lo}CD62L^{hi}) from *Senp3*$^{+/+}$*Cd4*-Cre and *Senp3*$^{fl/fl}$*Cd4*-Cre mice stimulated with anti-CD3 plus anti-CD28 antibodies and various concentrations (above plots) of TGF-β. Numbers above bracketed lines indicate percent Foxp3$^+$ cells. Data are representative of at least three independent experiments. Error bars are the mean ± SEM values. Two-tailed unpaired Student's *t* tests were performed. ns, not statistically significant.

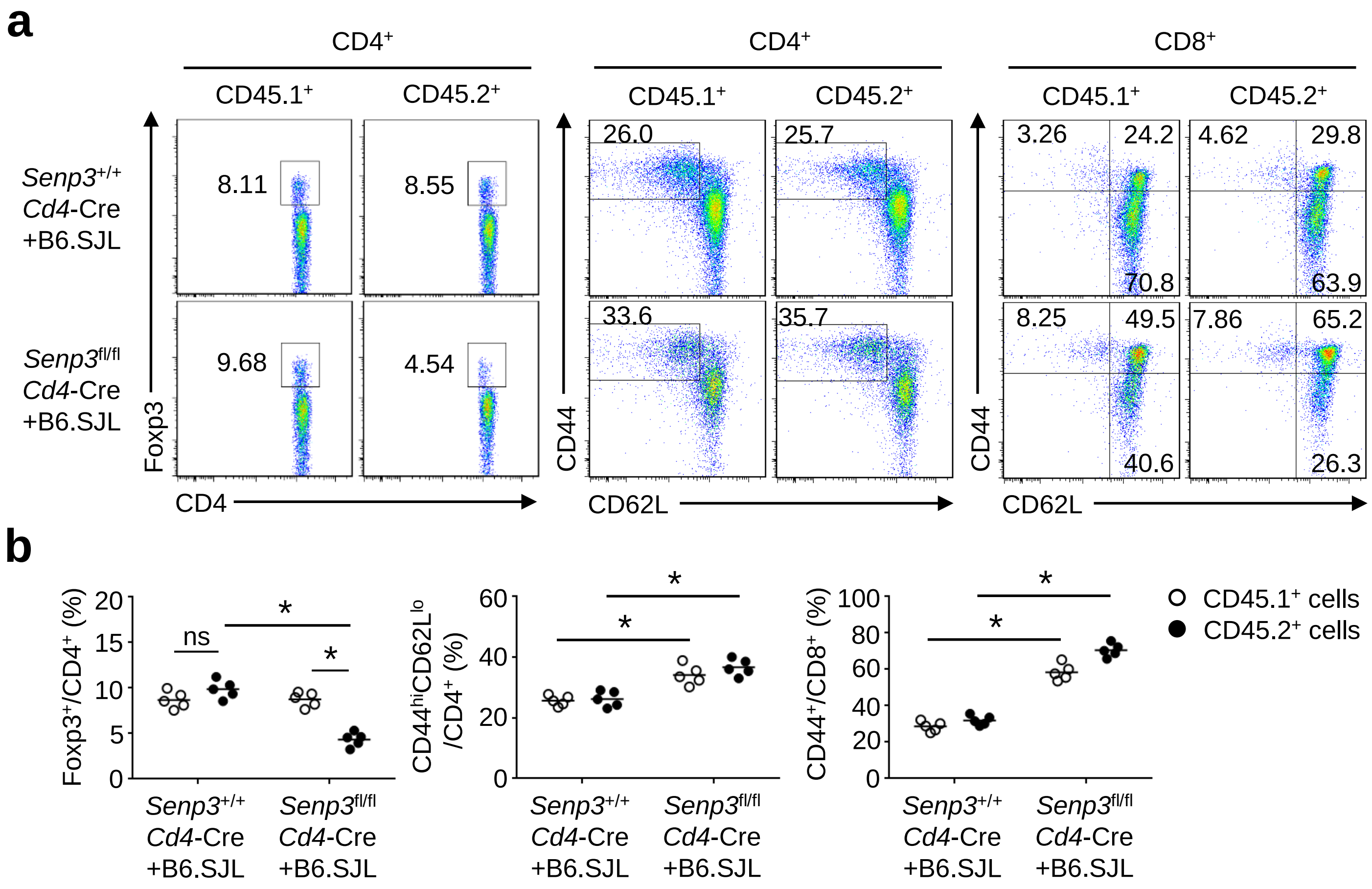

Supplementary Figure 4. Cell-intrinsic defect of SENP3-deficient Treg cell generation and function. (**a, b**) Flow cytometric analysis of splenocytes in *Rag1*$^{-/-}$ recipient mice adoptively transferred (for 8 weeks) with bone marrow (BM) cells derived from *Senp3*$^{+/+}$*Cd4*-Cre and *Senp3*$^{fl/fl}$*Cd4*-Cre mice (CD45.2^{+}) along with BM cells derived from B6.SJL mice (CD45.1^{+}), gating on CD45.1^{+} or CD45.2^{+} cells. Data are presented as representative plots (**a**) and summary graphs (**b**). Error bars are the mean ± SEM values. n = 5. Two-tailed unpaired Student's *t* tests were performed. ns, not statistically significant. *, P<0.05.

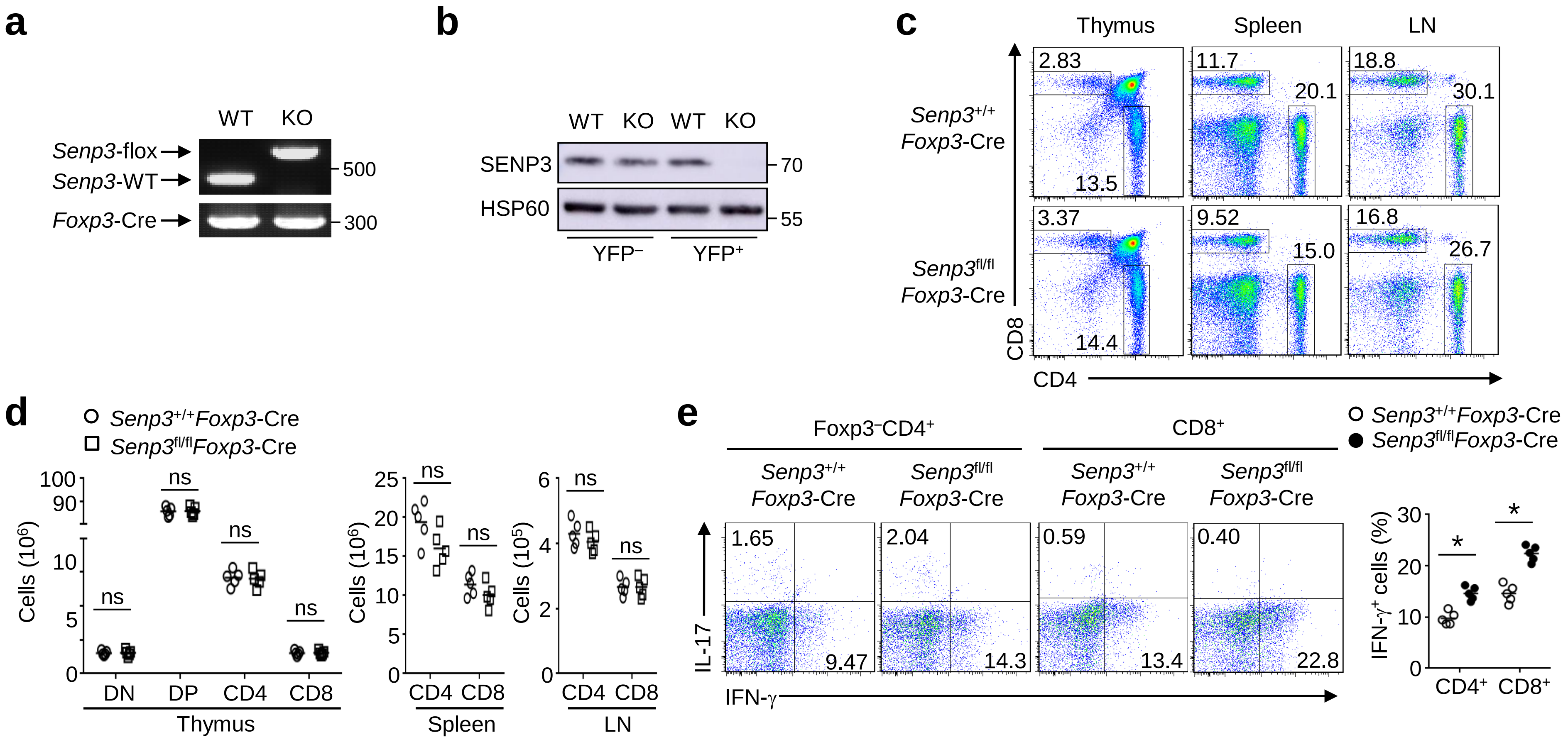

Supplementary Figure 5. Thymocyte development and peripheral T cell frequency in the *Senp3*$^{fl/fl}$*Cd4*-Cre mice. (**a**) Genotyping PCR to amplify the *Senp3*-flox and WT alleles (top) or the Cre cDNA (bottom). (**b**) Immunoblot analysis of SENP3, showing Treg cell–specific SENP3 ablation (WT: *Senp3*$^{+/+}$*Foxp3*-Cre; KO: *Senp3*$^{fl/fl}$*Foxp3*-Cre) . (**c**) Flow cytometric analysis of the percentage of CD4^{+} and CD8^{+} T cells from the thymus, spleen and lymph nodes (LN) of 6-week-old wild-type and *Senp3*$^{fl/fl}$*Foxp3*-Cre mice. (**d**) Cell number of CD4^{-}CD8^{-} (DN), CD4^{+}CD8^{+} (DP), CD4^{+}CD8^{-} (CD4), CD4^{-}CD8^{+} (CD8) in the thymus, and CD4^{+} and CD8^{+} T cells in the spleen and LN of 6-week-old wild-type and *Senp3*$^{fl/fl}$*Foxp3*-Cre mice. (**e**) Flow cytometric analysis of the percentage of IFN-γ-producing T cells in total splenocytes from 8-month-old wild-type and *Senp3*$^{fl/fl}$*Foxp3*-Cre mice. Data are representative of at least three independent experiments. Error bars are the mean ± SEM values. n = 5. Two-tailed unpaired Student's *t* tests were performed. ns, not statistically significant. *, P<0.05.

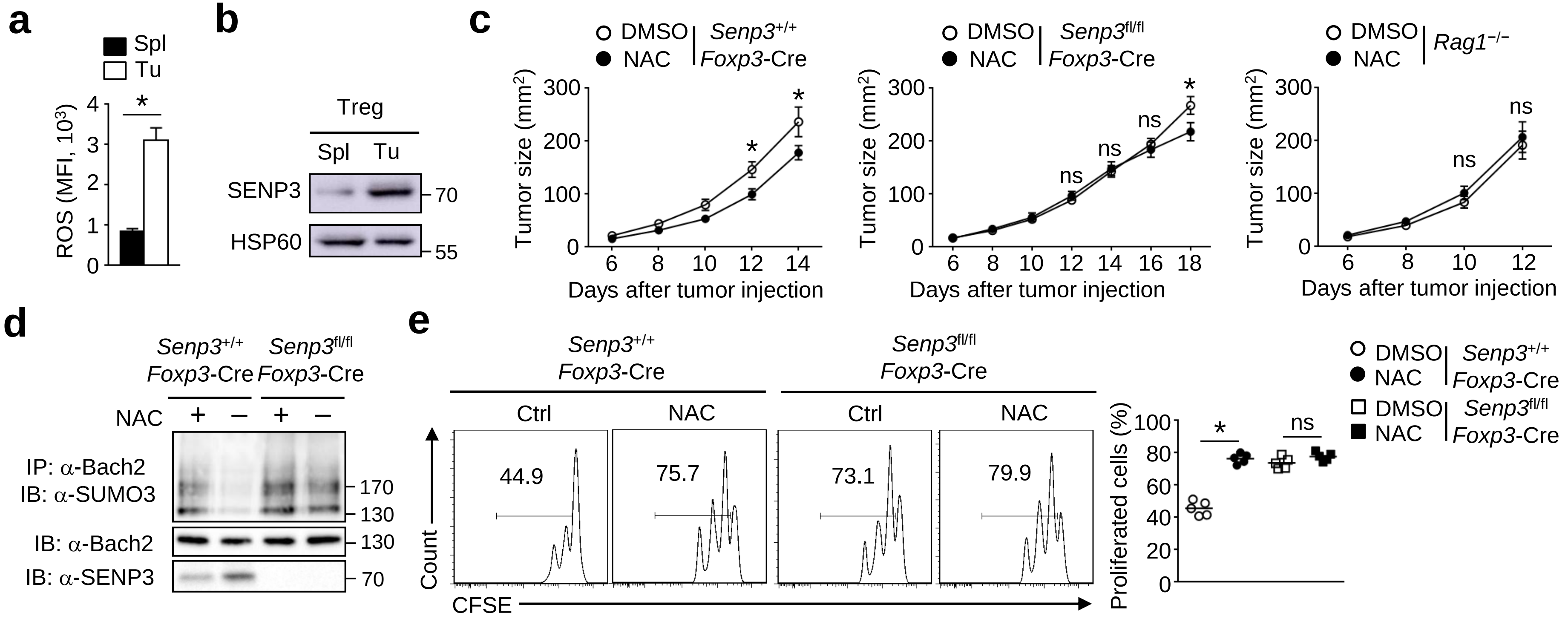

Supplementary Figure 6. NAC treatment promotes antitumor immunity and perturbs Treg cell function. (**a, b**) Levels of ROS (**a**) and SENP3 (**b**) in Treg cells from the spleen (Spl) and tumor (Tu) of tumor-bearing wild-type mice (MFI: mean fluorescence intensity). (**c**) Growth curve of tumors in 6-week-old *Senp3*$^{+/+}$*Foxp3*-Cre, *Senp3*$^{fl/fl}$*Foxp3*-Cre mice or *Rag1*$^{-/-}$ mice receiving daily NAC (n = 8 mice per group). (**d**) SUMOylation assays using tumor-infiltrating Treg cells from **c** (on day 14). (**e**) CFSE-labelled OT-I T cells were co-cultured with OVA peptide (257-264) and splenocytes from wild-type mice for 24 hours in the presence of tumor-infiltrating Treg cells from **c** (on day 14) at a ratio of 3:1 (OT-I/Treg cells). Data are representative of at least three independent experiments. Error bars are the mean ± SEM values. n = 5. Two-tailed unpaired Student's *t* tests were performed. ns, not statistically significant. *, P<0.05.

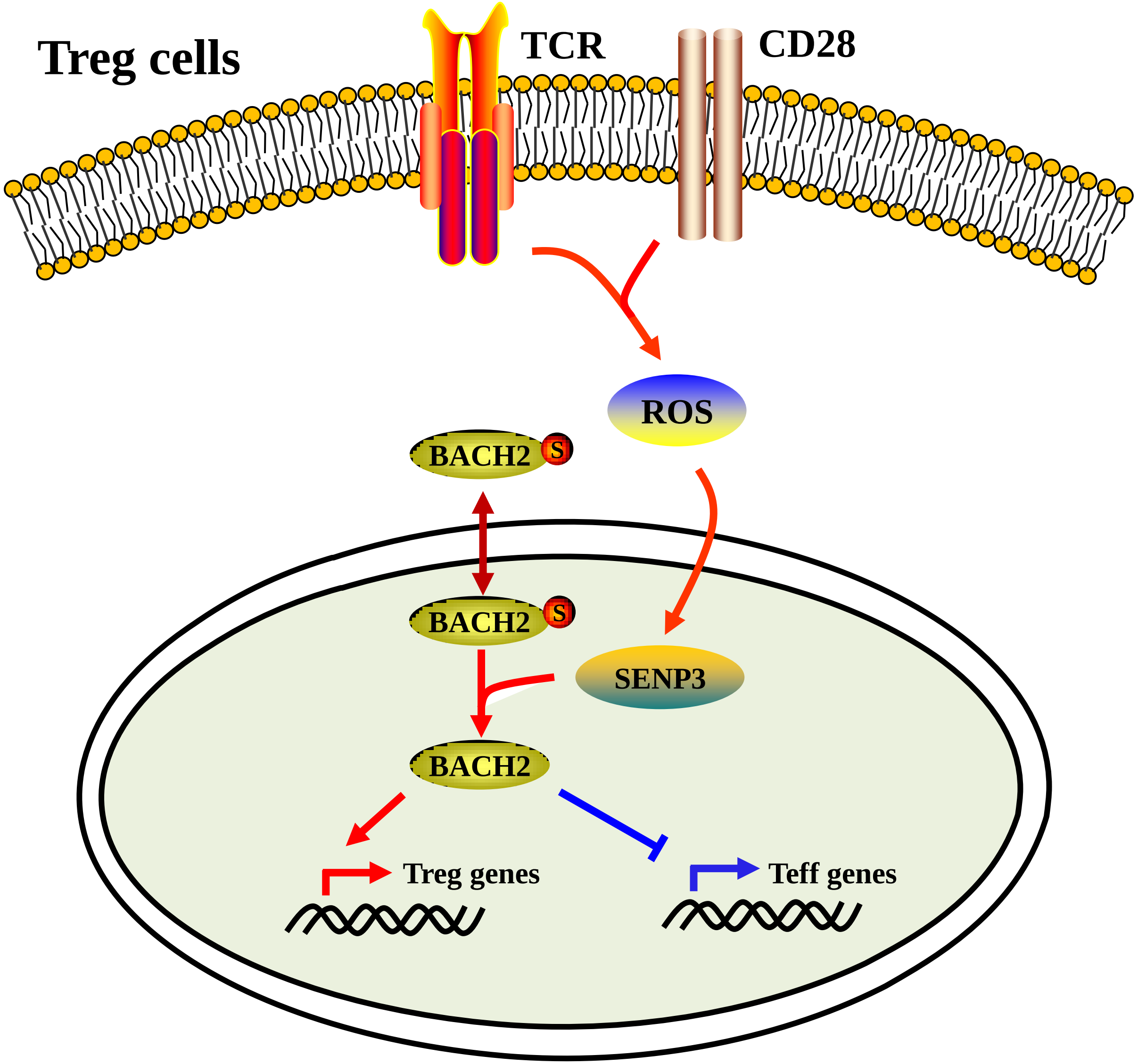

Supplementary Figure 7. SENP3 maintains the stability and function of regulatory T cells via BACH2 deSUMOylation. SENP3 is rapidly stabilized by TCR- and CD28-stimulated reactive oxygen species (ROS) in Treg cells. SENP3 accumulation triggered by ROS mediates BACH2 deSUMOylation to prevent its nuclear export, thereby repressing the genes associated with $CD4^+$ T effector cell differentiation and stabilizing Treg cell-specific gene signatures.

Fig. 5a

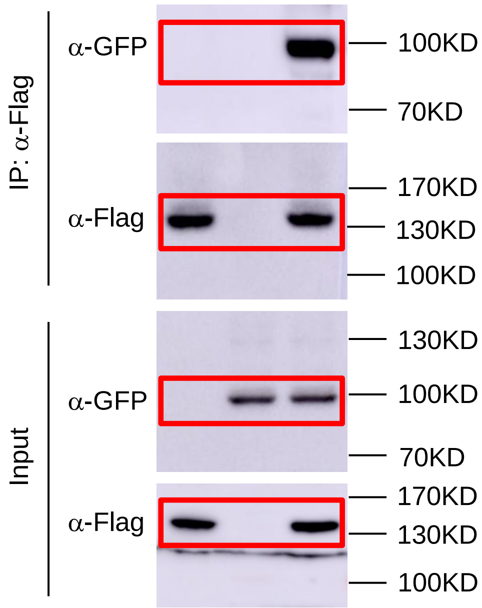

Fig. 5b

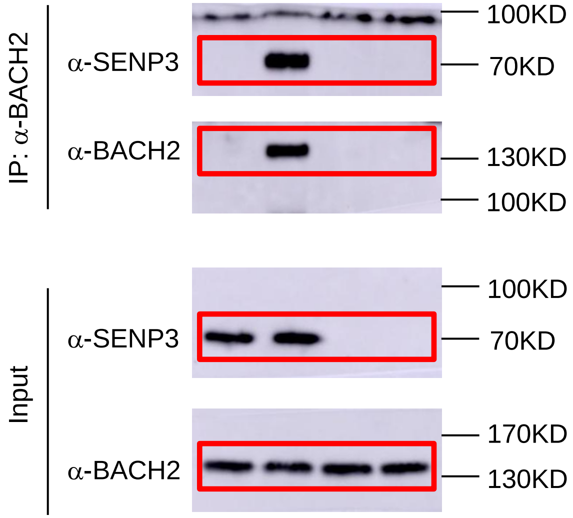

Fig. 5d

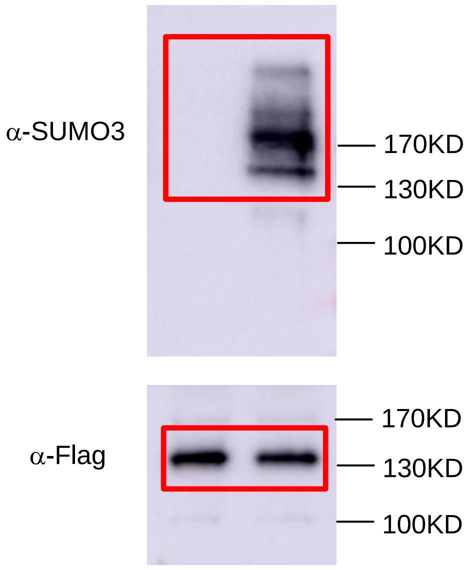

Fig. 5e

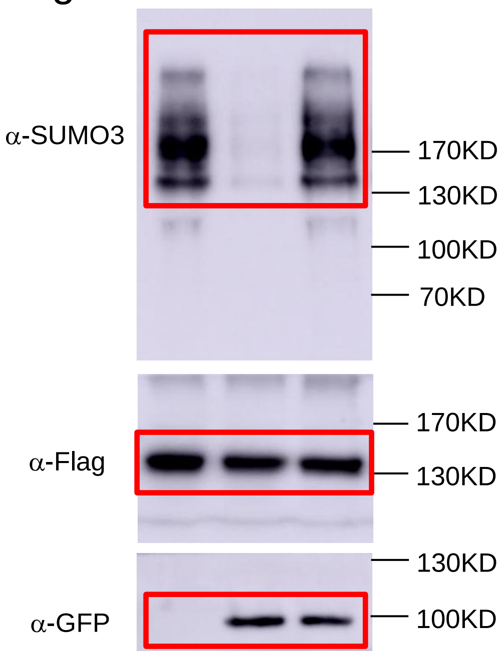

Fig. 5f

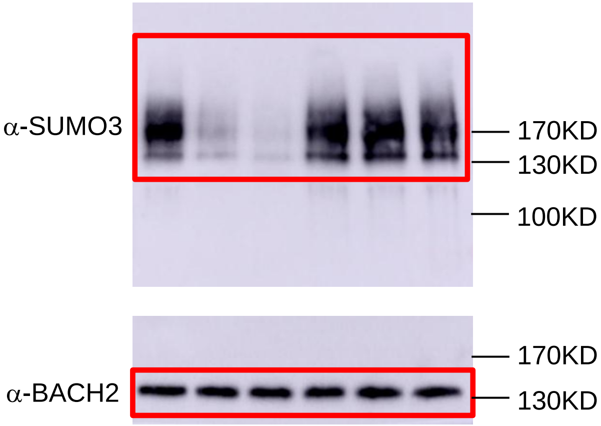

Fig. 5g

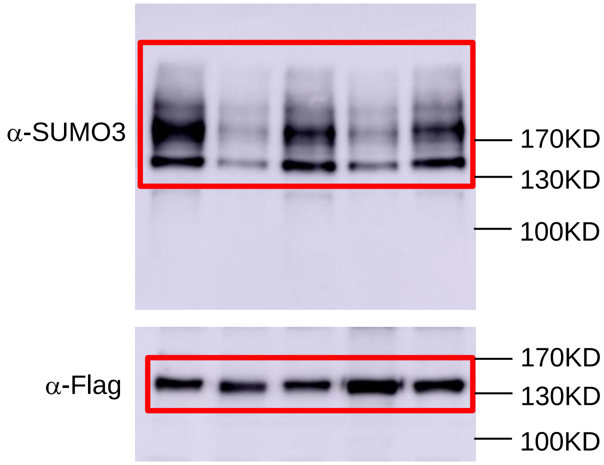

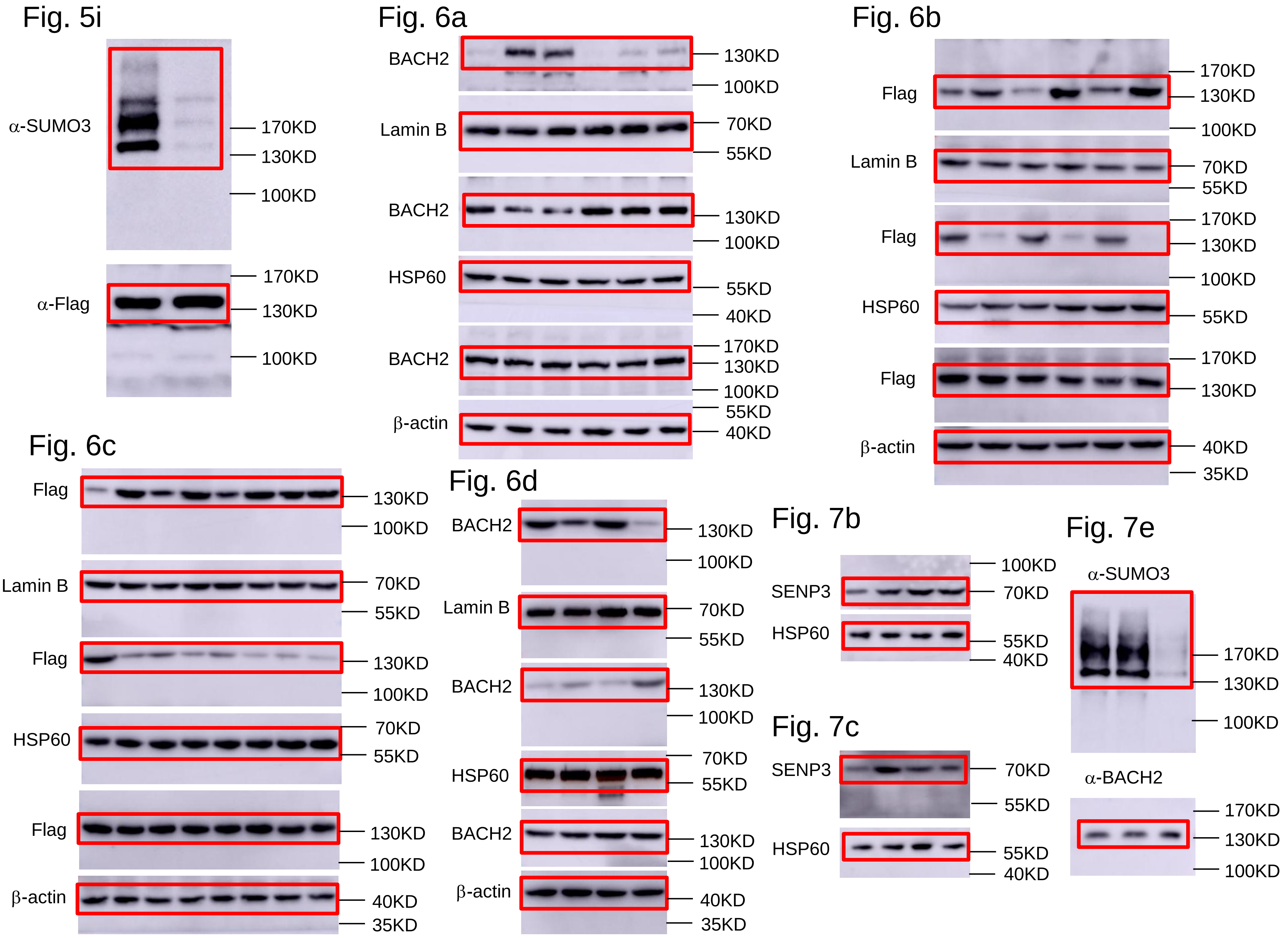

Supplementary Figure 8. All the uncropped scans of the western blots.

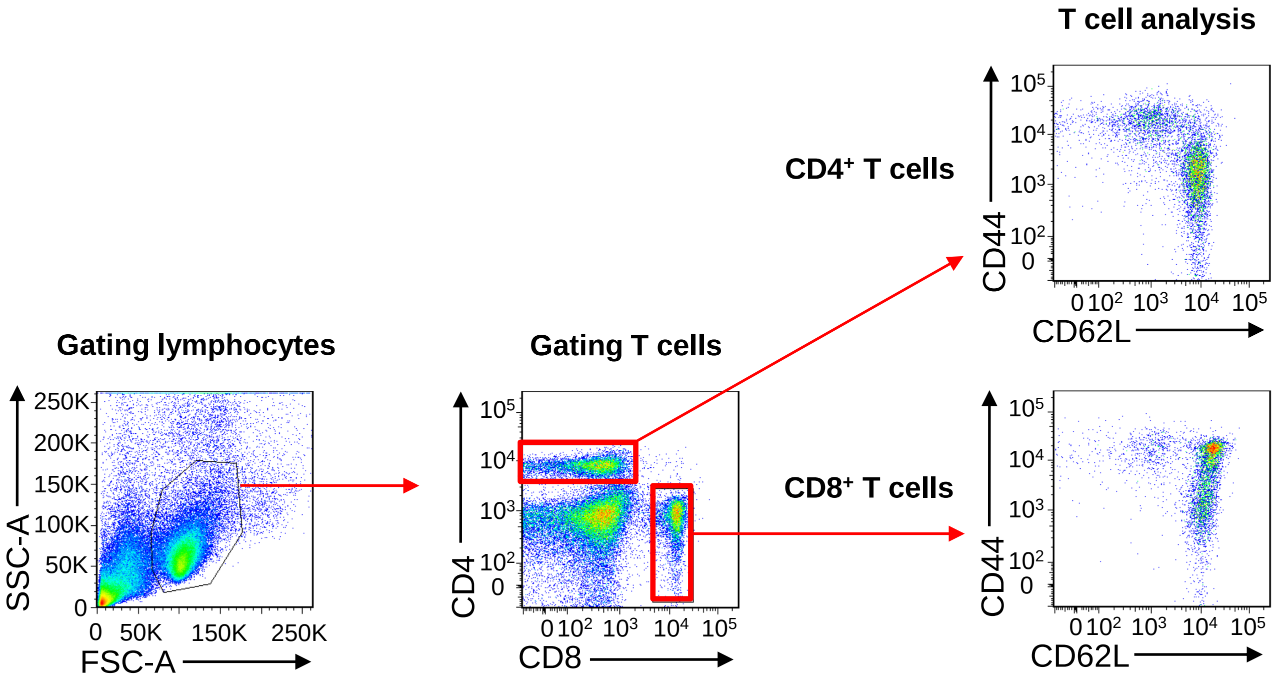

Supplementary Figure 9. All the FACS gating strategies.

Supplementary Table 1. Gene-specific primers used for qPCR.

Gene	Forward primers (5'->3')	Reverse primers (5'->3')
Ifng	CAGCAACAGCAAGGCGAAA	CTGGACCTGTGGGTTGTTGAC
Il13	CACACAAGACCAGACTCCCC	TCTGGGTCCTGTAGATGGCA
Il4	CGCCATGCACGGAGATG	CGAGCTCACTCTCTGTGGTGTT
Il21	AGGACCCTTGTCTGTCTGGT	GCTCACAGTGCCCCTTTACA
Il17a	AGCAGCGATCATCCCTCAAA	CTTCATTGCGGTGGAGAGTCC
Foxp3	ACTGGGGTCTTCTCCCTCAA	CGTGGGAAGGTGCAGAGTAG
Gpr83	CGCCCTTCACTTTGGTCATC	AGCGCACAATGTCCTCACTG
Gbp5	TTGTTTTTGACGCTCCTGCG	GGCTTTCTAGACGAGGTCCG
Actin	CGTGAAAAGATGACCCAGATCA	CACAGCCTGGATGGCTACGT

Supplementary Table 2. PCR primers used in ChIP-PCR.

Gene	Forward primers	Reverse primers
Il4	GAAGCAGGGATGGTCAGACT	TCCCCTAGCTATCCCCTAGC
Il5	GCTTCCCTCCCTTTCCAG	CCGACTTGGGGGTGAGTT
Ccr4	ATGTGGGGCTTCTTGACAAA	TCCCTGCCACAGAAGAAACT
Gapdh	CTACCCAAAAGGGACACCTACA	CATGACAACTTTGGCATTGTG
